# Analysis of the effect of an artificial intelligence chatbot educational program on non-face-to-face classes: a quasi-experimental study

**DOI:** 10.1186/s12909-022-03898-3

**Published:** 2022-12-01

**Authors:** Jeong-Won Han, Junhee Park, Hanna Lee

**Affiliations:** 1 College of Nursing Science, Kyung Hee University, 26 Kyunghee-Daero, Dongdaemun-Gu, Seoul, 02447 Republic of Korea; 2 College of Nursing Science, Dongnam Health University, 50, Cheoncheon-Ro 74Beon-Gil, Jangan-Gu, Suwon-Si, Gyeonggi-Do 16323 Republic of Korea; 3 Department of Nursing, Gangneung-Wonju National University, 150 Namwon-Ro, Heungeop-Myeon, Wonju-Si, Gangwon-Do 26403 Republic of Korea

**Keywords:** Artificial intelligence, Nursing, Education, Clinical reasoning, Chatbot program, Data processing

## Abstract

**Background:**

Education and training are needed for nursing students using artificial intelligence-based educational programs. However, few studies have assessed the effect of using chatbots in nursing education.

**Objectives:**

This study aimed to develop and examine the effect of an artificial intelligence chatbot educational program for promoting nursing skills related to electronic fetal monitoring in nursing college students during non-face-to-face classes during the COVID-19 pandemic.

**Design:**

This quasi-experimental study used a nonequivalent control group non-synchronized pretest–posttest design.

**Methods:**

The participants were 61 junior students from a nursing college located in G province of South Korea. Data were collected between November 3 and 16, 2021, and analyzed using independent t-tests.

**Results:**

The experimental group—in which the artificial intelligence chatbot program was applied—did not show statistically significant differences in knowledge (t = -0.58, *p* = .567), clinical reasoning competency (t = 0.75, *p* = .455), confidence (t = 1.13, *p* = .264), and feedback satisfaction (t = 1.72, *p* = .090), compared with the control group; however, its participants’ interest in education (t = 2.38, *p* = .020) and self-directed learning (t = 2.72, *p* = .006) were significantly higher than those in the control group.

**Conclusion:**

The findings of our study highlighted the potential of artificial intelligence chatbot programs as an educational assistance tool to promote nursing college students’ interest in education and self-directed learning. Moreover, such programs can be effective in enhancing nursing students’ skills in non-face-to face-situations caused by the ongoing COVID-19 pandemic.

## Introduction

Along with the development of technology, such as big data, machine learning, and artificial intelligence (AI), intelligent services have been actively introduced in the field of information technology. AI has been initiated in clinical practice and nursing education because of the restrictions in education caused by the coronavirus disease (COVID-19) pandemic [1]. Moreover, conventional lessons have been converted into video lectures and non-face-to-face lessons. Thus, strategies for improving students’ self-directed learning, and efforts for promoting interactions between instructors and students are needed. This has led to a growing interest in using chatbots in the education field. A chatbot—also referred to as “a talking bot”—is a type of a software prominently emerging in the information technology field [2], that can have verbal or written conversations with human users, and address their requests, using the question-and-answer format [3].

A chatbot also has various applications in the education field, as people can use it to learn without time and space restrictions [4]. It also improves the effect of self-directed learning as learners experience low levels of stress while engaging in conversations with a chatbot and repeated learning [5]. Furthermore, it facilitates immediate user feedback through conversations during the learning process, and provides customized contents based on the feedback [6]. Therefore, through AI technology, a chatbot can provide education to those who are unable to seek help from instructors because of problems, such as cost, manpower, and the COVID-19 pandemic [1, 7].

## Background

Electronic fetal monitoring (EFM) involves attaching a device to the abdominal wall of pregnant women to continuously monitor and record fetal heart sounds through graphs. It is utilized to prevent fetal hypoxia and provide interventions at an early stage by observing changes in fetal heartbeat [8]. As a noninvasive method to assess fetal health, EFM is widely used in the obstetrics field [8]. However, it is difficult to interpret the graphs, which have been found to have low specificity, compared with the level of sensitivity [9]. Thus, healthcare providers and nursing students should be able to accurately install the device, interpret the graphs, and report abnormal patterns to the doctors.

Regarding essential nursing techniques in maternal health nursing, education on installing EFM equipment and interpreting its results is required [10]. Additionally, during their training in the delivery room, nursing students learn about measuring vital signs (57.5%), supportive touch in the first stage of labor (42.7%), patient transport (41.7%), securing patient privacy (38.1%), followed by interpretation of EFM results (33.1%) [11]. Since EFM-related tasks, such as comprehending the correlation between fetal heartbeat and pressure in the womb, require professional knowledge and understanding, nursing students should be provided with sufficient learning and training in EFM prior to their training in the delivery room [12].

In maternal health nursing, the learning goals associated with EFM are “explanation of the purpose and method of EFM,” “understanding of EFM results,” “purpose of the nonstress test and manipulation of the machine,” and “performance of nursing techniques upon fetal asphyxia.” These goals underline the expectations for nursing students, including to understand the purpose, method, and principles of EFM, and be equipped with the skills to perform EFM and interpret its results [13]. However, previous studies show that only supplementary materials for understanding and performing EFM are utilized in self-directed learning [12], and education on high fidelity simulation is provided, both of which, are inadequate for developing overall nursing skills [14], thus highlighting the need for various educational methods for nursing students’ education and training.

In today’s society, where high-level knowledge and clinical judgment skills are required and evidence for clinical application is fast-changing, the importance of educational programs utilizing AI, that allow safe and efficient learning for nursing college students has been emphasized [1]. Therefore, applications of nursing education utilizing AI are needed. To date, studies regarding chatbots in the medical field have focused on its application in the anatomy class for medical students [15] and chatbot programs for managing mental health, learning achievement, and well-being of college students [16]. In particular, in a study targeting 4th grade medical students in Hong Kong, clinical history was obtained by talking with a virtual patient through a chatbot mobile app without time or geographical restrictions. Based on the results of this study, it is suggested that chatbot programs can be an alternative to the existing clinical practice methods [17]. However, few studies have assessed its effect in nursing education. In other educational fields, using chatbot has shown improvements in students’ knowledge, academic motivation, and learning satisfaction [18]. Therefore, developing chatbot educational programs and evaluating their effects in the field of nursing education are needed. In this study, we aimed to develop and evaluate the effect of an AI chatbot educational program for improving nursing college students’ EFM nursing skills.

### Research hypotheses

1. There will be differences in the EFM knowledge between the experimental group, which participated in the AI chatbot educational program, and the control group.
2. There will be differences in the clinical reasoning competency for EFM between the experimental group and the control group.
3. There will be differences in the confidence in assessing fetal health between the experimental group and the control group.
4. There will be differences in the interest in education between the experimental group and the control group.
5. There will be differences in the self-directed learning between the experimental group and the control group.
6. The experimental group will have higher feedback satisfaction than the control group.

## Methods

### Study design

This quasi-experimental study used a nonequivalent control group pretest–posttest design for developing and assessing the effect of an AI chatbot educational program for non-face-to-face video lectures on EFM for nursing college students.

### Study participants

**Fig. 1:**
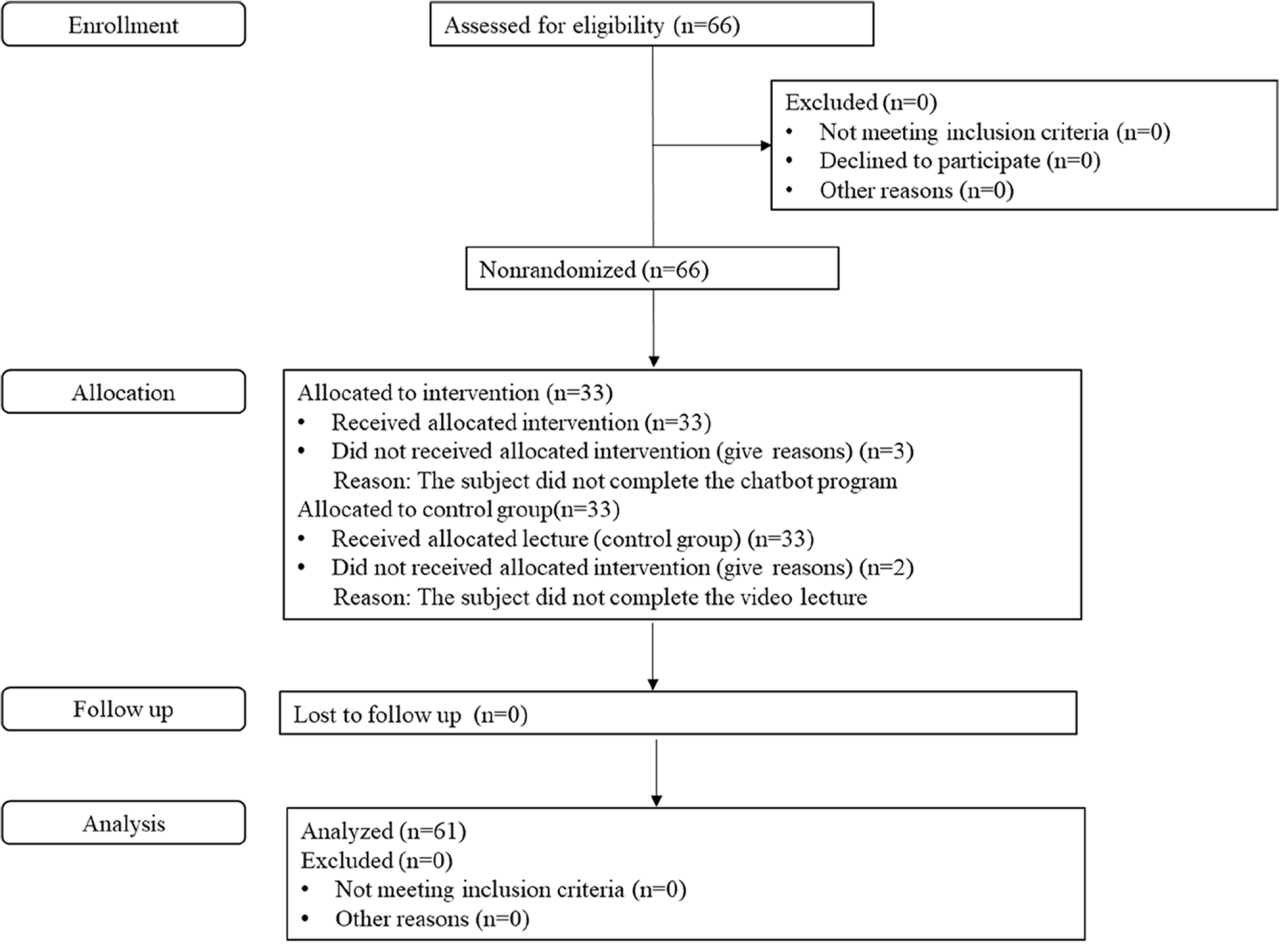
Study participant

The participants were junior students at the university of nursing located in G province of South Korea. Their selection criteria were as follows: 1) nursing students, 2) voluntary participation, and 3) no experience of an EFM educational program utilizing chatbot. The exclusion criteria were nurses or students with nurse’s aide certification, as those with this certification would have prior knowledge obtained from their clinical experience, which might have interfered with this study’s assessment. The sample size was calculated using G*Power version 3.1.9.2 [19]. The minimum sample size for each of the two groups was calculated as 26, based on the two-tailed test of the difference between two independent means with a 1:1 ratio, test power of 0.80, significance level of 0.05, and effect size of 0.80. Considering a 20.0% dropout rate, 66 participants (33 in each group) were selected. Since the data of three participants of experimental group who did not complete the chatbot program were excluded and two participants of control group who did not complete the video lecture were excluded. The final number of participants in the control and experimental groups were 31 and 30, respectively (Fig. 1). Since previous studies that assessed the effect of using chatbot in college students’ education had an effect size larger than 0.8 [7, 20], the effect size in this study was set at 0.8, which corresponds to a large effect size, as presented by Cohen [21].

### Study stages

In the analysis stage, nursing college students’ requests regarding the function and contents of the chatbot program were analyzed, and a literature search for EFM nursing education was conducted to develop the contents and learning goals of the AI chatbot educational program for EFM.

In the design stage, the program’s process and service interface were designed, using an algorithm that allows customized interventions to be provided on the platform. Furthermore, the service interface was designed to increase the study participants’ readability and concentration.

**Fig. 2:**
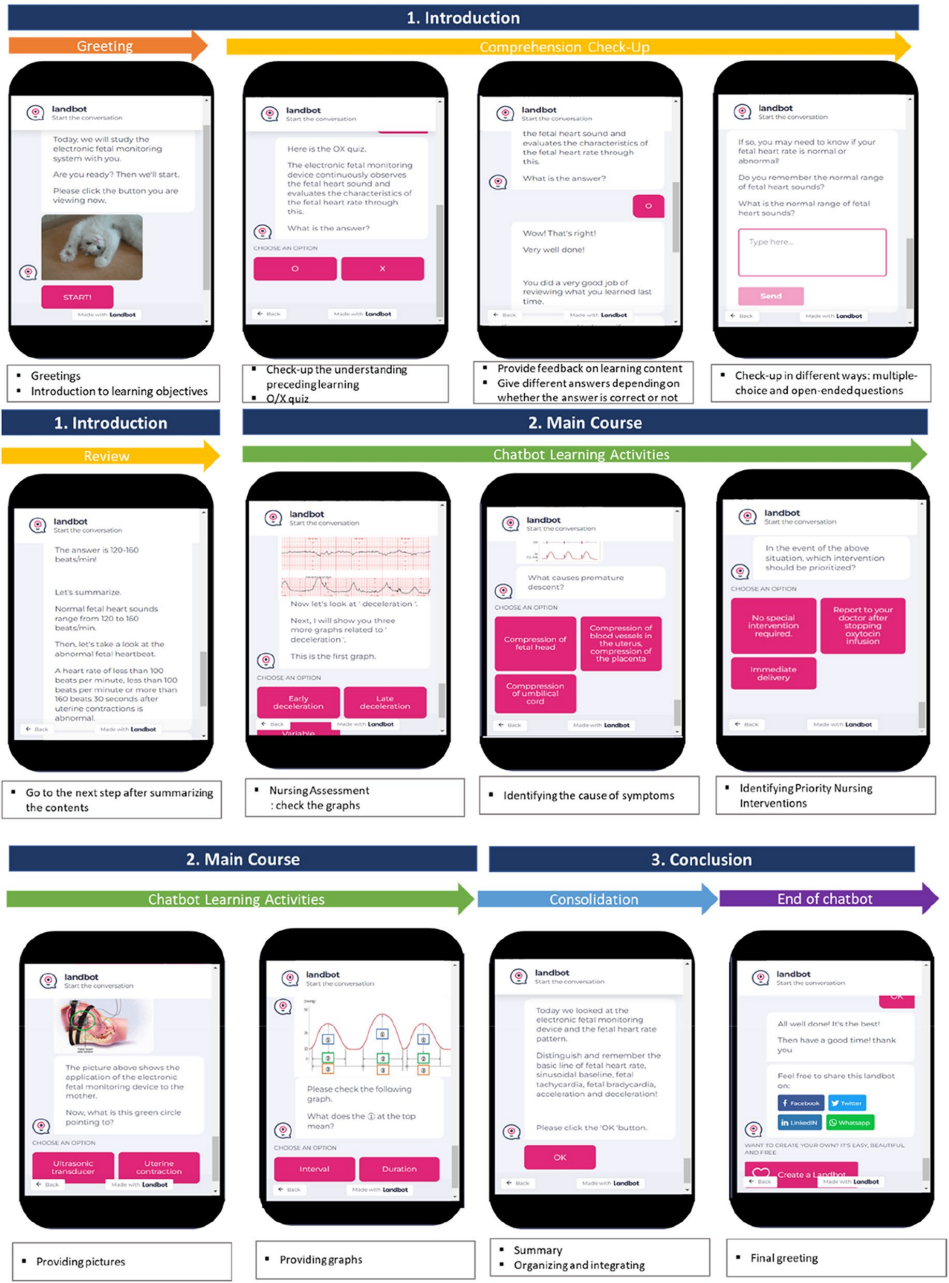
Consists of chatbot program

The user interface was designed using LandBot.io (https://landbot.io/). Users can enter questions and see the answers in the chatbot through the user interface. When a user enters a question in a natural language, the intention and entities of the question are recognized by the natural language processing engine, following which, the most adequate answer is selected and provided to the user from a database of accumulated learning results. The chatbot consists of introduction, main course, and conclusion stages (Fig. 2). In the introduction stage, students are first greeted and introduced to the learning objectives of the chatbot program. The next step is to check-up the understanding of video preceding learning. Various methods such as O/X quiz, multiple choice, and open-ended questions are used to check the understanding of preceding learning. Feedback is provided after every question, and it is different depending on whether the answer is correct or not. If the answer is not correct, the contents of the study are rearranged so that the students can study again. After the check-up of the preceding learning is completed, the contents are summarized and then students can proceed to the next step. In the main course, students learn about nursing management and nursing interventions of electronic fetal monitoring devices through chatbot learning activities. Students read and interpret related graphs, identify patient symptoms, and learn to prioritize nursing interventions accordingly. In addition, students can experience equipment through various pictures and photos. Chatbot learning activities also provide feedback based on students’ responses and enhance their learning experience. Finally, in the conclusion section, students organize and integrate what they have learned so far. Then, the chatbot program ends with a final greeting.

Moreover, the heuristics and performance of the program were evaluated by experts and modified and adjusted accordingly. Thereafter, the chatbot was used by nursing college students, whose user experiences were evaluated, and the performance of the chatbot was revalidated.

### Study instruments

The participants’ knowledge of EFM was assessed using 13 questions as follows: three questions on understanding and explaining the purpose and method of EFM, seven questions on examining the results of EFM, and three questions on knowledge of nursing interventions based on the EFM results. With one point for every correct answer, the total scores ranged from 0‒20, with higher scores indicating better knowledge of EFM. The content validity was verified by four experts (three nursing professors who had teaching experience in women’s health, and nursing science, and a nurse with more than 10 years of experience in the delivery room), and only those items with a content validity index of 0.8 or higher were selected and finalized. The reliability of the instrument was calculated as 0.79 using Kuder-Richardson Formula 20.

In this study, clinical reasoning competency were measured with 15 questions developed by Liou et al. [22], using a 5-point Likert scale, and translated and validated by Joung and Han [23]. Higher scores indicate a higher level of clinical reasoning competency. The reliability of the instrument was Cronbach’s α = 0.94 in Liou et al.’s study [22], and Cronbach’s α = 0.93 in Joung and Han’s study [23]. In this study, the reliability of the instrument was Cronbach’s α = 0.96.

Furthermore, confidence in fetal health assessment using EFM was measured using three questions. For each question, a response of “strongly confident” and “not confident at all” accounts for 10 and 0 points, respectively. A higher total score indicates a higher level of confidence. The reliability of the instrument was Cronbach's α = 0.91.

In this study, interest in education, assistance for self-directed learning, and feedback satisfaction were measured using numerical rating scales. For each question, assessing interest in education, a response of “strongly confident” and “not confident at all” accounts for 10 and 0 points, respectively. A higher total score indicates a higher level of interest in education. Moreover, for each question assessing assistance for self-directed learning, a response of “very helpful” and “not helpful at all” account for 10 and 0 points, respectively. A high score indicates a high level of self-directed learning Finally, for each question assessing feedback satisfaction, a response of “very satisfied” and “not satisfied at all” account for 10 and 0 points, respectively. A higher total score indicates a higher level of feedback satisfaction.

### Data collection

Data were collected between November 3 and 16, 2021. Due to the recurrence of COVID-19, this study was conducted using non-face-to-face video lectures. The experimental and control groups completed an online pre-test questionnaire prior to the commencement of the video lectures. The experimental group attended both the video and chatbot lectures, whereas the control group only attended the video lectures. A video lecture was approximately 32 min long with a professor delivering a unidirectional lecture without obtaining feedback. The learning goals of these video lectures were as follows: 1) explaining the purpose and method of EFM, 2) interpreting the results of EFM during labor, 3) understanding the purpose of the nonstress test and performing it by manipulating the device, 4) explaining the purpose, method, and results of the contraction stress test, and 5) applying nursing procedures in the presence of fetal distress. The control group submitted the post-test questionnaires online after the video lectures, following which, they were allowed to attend the chatbot lectures. Meanwhile, the experimental group submitted the post-test questionnaires after the video and chatbot lectures.

### Statistical analysis

Collected data were analyzed using SPSS/WIN 23. A Shapiro–wilk test was performed to test the normality of variables before applying the program. Pre-test homogeneity testing of the participants’ general characteristics and measurement variables was performed using chi-squared tests, Fisher’s exact tests, and t-tests. After the intervention, independent t-tests were performed to compare the differences in knowledge, clinical reasoning competency, interest in education, self-directed learning, and feedback satisfaction between the experimental and control groups.

### Ethical considerations

This study was conducted after obtaining an approval from the Institutional Review Board of Dongnam Health University (1044371–202109-HR-006–01). Instructions on study participation and a consent form were attached to the questionnaire, and data were collected after explaining the study to the participants. The consent form provided information regarding voluntary participation, assurance of confidentiality, and the scope of the application of the study’s results. Moreover, the participants were assured that they could withdraw participation at any time and it would not affect their grades. Additionally, they were informed that the program would not invade their privacy.

## Results

### Participants’ general characteristics

The participants’ general characteristics are presented in Table 1. The control and experimental groups had 31 (50.9%) and 30 (49.1%) participants, respectively, comprising 5 (8.2%) and 56 (91.8%) men and women, respectively. Fourteen (23.0%) participants reported being religious and 47 (77.0%) irreligious. Regarding grades in the previous semester, 11 (18.0%), 37 (60.7%), and 13 (21.3%) participants scored less than 3.0, 3.0‒3.5, and 3.5 or higher, respectively. Regarding satisfaction with college life, 33 (54.1%), 21 (34.4%), and 7 (11.5%) participants responded “satisfied,” “somewhat satisfied,” and “not satisfied,” respectively. In terms of satisfaction with their majors, 37 (60.7%), 19 (31.1%), and 5 (8.2%) participants responded “satisfied,” “somewhat satisfied,” and “not satisfied,” respectively. Finally, regarding interest in women’s health and nursing science, 41 (67.2%), 18 (29.5%), and 2 (3.3%) participants responded “interested,” “somewhat interested,” and “not interested,” respectively. Homogeneity testing for general characteristics revealed no statistically significant differences between the two groups.

**Table 1:** Homogeneity Test of General Characteristics of Subjects (*N* = 61)

Variables	Category	Control (*n* = 31) n (%)	Experimental (*n* = 30) n (%)	Total (*N* = 61) n (%)	χ^2^/t	*P*-value
Gender	Male	4 (6.6)	1 (1.6)	5(8.2)	-	.187^†^
	Female	27 (44.3)	29 (47.5)	56(91.8)		
Religion	Yes	9 (14.8)	5 (8.2)	14(23.0)	1.32	.251
	No	22 (36.1)	25 (41.5)	47(77.0)		
Grade of last semester (5.0 point scale)	< 3.0	6(9.8)	5(8.2)	11(18.0)	1.01	.603
	3.0―3.5	17(27.9)	20(32.8)	37(60.7)		
	≥ 3.5	8(13.1)	5(8.2)	13(21.3)		
Satisfaction with their university life	Satisfied	21 (34.4)	12 (19.7)	33(54.1)	4.916	.086
	Somewhat satisfied	7 (11.5)	14 (23.0)	21(34.4)		
	Not satisfied	3(4.9)	4(6.6)	7(11.5)		
Satisfaction with their major	Satisfied	21 (34.4)	16 (26.2)	37(60.7)	2.18	.337
	Somewhat satisfied	7(11.5)	12(19.7)	19(31.1)		
	Not satisfied	3 (4.9)	2 (3.3)	5(8.2)		
Interest in Maternal Nursing	Interested	23 (37.7)	18 (29.5)	41(67.2)	2.82	.245
	Somewhat interested	8(13.1)	10(16.4)	18(29.5)		
	No interested	0 (0.0)	2 (3.3)	2(3.3)		

^†^Fisher’s exact test

### Homogeneity testing

Homogeneity testing for the participants’ measurement variables showed no statistically significant differences between the two groups (Table 2).

**Table 2:** Homogeneity Test of Dependent Variables (*N* = 61)

Variables	Group	M ± SD	Skewness	Kurtosis	t	*P*-value
Knowledge	Control	6.00 ± 1.95	-.152	-.339	-0.494	.155
	Experimental	5.70 ± 2.77	.585	-.060		
Clinical reasoning competency	Control	48.42 ± 9.25	.517	1.425	0.167	.868
	Experimental	48.80 ± 8.50	-.992	2.431		
Confidence	Control	16.65 ± 5.57	-.95	1.480	-0.321	.749
	Experimental	16.20 ± 5.26	-.568	-.181		
Interest in education	Control	6.87 ± 2.08	-.460	-.655	-1.459	.150
	Experimental	6.07 ± 2.23	-.069	-1.287		
Self-directed learning	Control	7.06 ± 1.88	-.099	-.696	-1.208	.233
	Experimental	6.57 ± 2.11	-.417	-.123		

*M* Mean, *SD* Standard deviation, *SE* Standard error

### Hypothesis testing

The test results of the differences in measurement variables between the experimental and control groups are presented in Table 3. The experimental group, in which, the AI chatbot educational program for EFM was applied, did not show statistically significant differences in knowledge (t = -0.58, *p* = 0.567), Clinical reasoning competency (t = 0.75, *p* = 0.455), confidence in fetal health assessment (t = 1.13, *p* = 0.264), and feedback satisfaction (t = 1.72, *p* = 0.090), compared with the control group. However, participants in the experimental group showed significantly higher interest in education (t = 2.38, *p* = 0.020) and self-directed learning (t = 2.72, *p* = 0.006) than those in the control group.

**Table 3:** Effects on Education Program (*N* = 61)

Variables	Group	Pre-test M ± SD	Post-test M ± SD	Mean differences (post–pre) M ± SD	*t*	*P*
Knowledge	Exp	5.70 ± 2.77	10.07 ± 2.24	4.37 ± 2.88	-0.58	.567
	Con	6.00 ± 1.95	10.77 ± 1.70	4.77 ± 2.63		
Clinical reasoning competency	Exp	48.80 ± 8.50	60.70 ± 7.14	11.90 ± 6.91	0.75	.455
	Con	48.42 ± 9.25	58.97 ± 8.74	10.54 ± 7.12		
Confidence	Exp	16.20 ± 5.26	23.10 ± 3.82	6.90 ± 5.6	1.13	.264
	Con	16.65 ± 5.57	21.90 ± 4.70	5.26 ± 5.77		
Interest in education	Exp	6.07 ± 2.23	8.40 ± 1.43	2.17 ± 2.60	2.38	.020
	Con	6.87 ± 2.08	7.77 ± 1.77	0.09 ± 1.37		
Self-directed learning	Exp	6.57 ± 2.11	8.77 ± 1.19	2.20 ± 2.43	2.72	.006
	Con	7.06 ± 1.88	7.87 ± 1.62	0.81 ± 1.47		
Satisfaction with feedback	Exp		8.67 ± 1.24		1.72	.090
	Con		8.03 ± 1.68			

*M* Mean, *SD* Standard deviation, Experimental, *Con* Control

## Discussion

This study aimed to develop and evaluate the effect of an AI chatbot educational program for improving nursing college students’ EFM nursing skills. First, although the post-test scores in both experimental and control groups showed an increase in participants’ knowledge of EFM, compared with the pre-test scores, the difference was not statistically significant between the two groups. This result is similar to that of a previous study that examined the effect of an AI chatbot used in fifth-grade science classes [6], where no difference was found between the experimental and control groups’ academic performance. However, another study reported that learners’ knowledge was enhanced by using a chatbot for educational purposes [18]. In our study, since both the experimental and control groups attended the same video lectures, which contained detailed information on the purpose and method of EFM, interpretation of results, examples of EFM graphs, etc., it could have been the reason behind the participants’ improvement in knowledge of EFM in both the experimental and control groups. Nonetheless, a chatbot program allows immediate corrections when incorrect knowledge is presented by nursing students, and offers customized contents based on the feedback received, making it effective for acquiring complex nursing knowledge. However, its effect on knowledge needs to be further verified in future studies.

Second, there were no significant differences in clinical reasoning competency and confidence in fetal health assessment using EFM between the two groups. Although an accurate comparison cannot be made due to lack of studies measuring clinical reasoning competency and fetal health assessment in nursing college students using chatbot, the measurement tool used for assessing clinical reasoning competency in this study comprised questions related to the level of knowledge, attitude, and techniques required to determine normal or abnormal conditions of patients and performing nursing interventions accordingly. Nonetheless, there might have been restrictions in using this tool in our study, which aimed to evaluate the effect of a chatbot educational program. Moreover, previous studies [6, 18, 24] have evaluated chatbot programs by assessing academic engagement and participation. Thus, long-term effects resulting from increased academic engagement and participation, such as an increase in clinical reasoning competency or changes in performance, need to be examined further.

Third, there was a significant difference in the participants’ interest in education between the experimental and control groups. This result is in line with that of a study that found improved academic motivation in learners who used a chatbot [18], and another one that examined the effect of an AI chatbot, which reported that using a chatbot had a positive effect on the experimental group students’ online learning experience [6]. Furthermore, Deveci [6] described students’ experience of using chatbot as “useful and fun, wanting to use it in other classes, useful for learning outside of classes, and classes can be repeated.” Our study also demonstrated that using a chatbot program increased the participants’ interest in education, and it was considered useful by them. As such, a chatbot program can positively affect students’ learning during the COVID-19 pandemic, and its application is likely to increase nursing students’ interest in education and positively contribute toward their learning experiences.

Fourth, there was a significant difference in the level of self-directed learning between the experimental and control groups. This is similar to the result of a previous study, that used a 15-week chatbot program for students majoring in computer science, which reported that it assisted in self-directed learning since 72% of the students showed improved participation in subjects [24]. Regarding convenience, the AI chatbot educational program developed in our study allowed easy access to learning contents through the internet and a mobile device, regardless of time and space, enabling ubiquitous learning. Hence, smartphones equipped with a chatbot can be used as a mobile learning tool that allows immediate responses and promotes human interactions, without restrictions of time and space [17, 25], thereby facilitating students’ self-directed learning. Moreover, as the chatbot used in our study asked questions based on the students’ learnings from video lectures and offered feedback based on their answers, it provided a learning environment that allowed students to discover what they had missed and relearn accordingly. This suggests that using an AI chatbot program will be helpful for promoting self-directed learning in nursing students.

Finally, no significant difference in feedback satisfaction was found between the experimental and control groups. This is in contrast to the results of a previous study that used chatbot in education [18], which reported that the chatbot improved learners’ academic satisfaction. Furthermore, it contradicts the finding that a chatbot is effective as it enables immediate real-time feedback and can correct students’ errors [4, 6]. This result might have been due to the limitations of our study’s chatbot, that was a type of transaction chatbot, which provided feedback based on predicted responses, but did not address specific questions asked by the students. However, along with the development of data processing and AI technology, computer programs using chatbots are able to understand and answer questions similar to humans [15]. Therefore, application of conversation-type or hybrid chatbot technology in future studies could possibly improve students’ feedback satisfaction.

## Conclusions

In this study, we developed and evaluated an AI chatbot educational program to enhance EFM nursing skills in nursing college students. Our findings hold significance as they present the potential of information and communications technology applications, such as chatbot, in the nursing education field. The chatbot program developed in our study had positive effects on the nursing college students’ interest in education and self-directed learning. Therefore, it can serve as an innovative and effective educational tool for improving students’ nursing skills, especially in non-face-to-face situations caused by the ongoing COVID-19 pandemic. However, our study only evaluated the effect of the program in participants from only one college and selecting a nonequivalent study design. As a post-test round was conducted on measurement variables in this study, we propose future studies with longitudinal design evaluating medium or long-term effects.

## Data Availability

The datasets used and analyzed during the current study are available from the corresponding author on request.

## Abbreviations

AI: Artificial Intelligence
COVID-19: Coronavirus Disease of 2019
EFM: Electronic fetal monitoring
